# Comprehensive Analysis of Malate Accumulation in Peaches in Response to Cold Stress Based on Transcriptomics

**DOI:** 10.1002/fsn3.71425

**Published:** 2026-01-07

**Authors:** Hongfang Cai, Shuai Han, Xu Wang, Jiqing Shi, Zewen Xiong, Zheng Zhang, Zhuyi Gao

**Affiliations:** ^1^ College of Biology and Food Engineering Suzhou University of Technology Changshu P.R. China; ^2^ School of Food and Pharmacy Shanghai Zhongqiao Vocational and Technical University Shanghai P.R. China; ^3^ Suzhou Shenyuan Bio‐Tech Co., Ltd. Suzhou P.R. China

**Keywords:** chilling injury, malate accumulation, *Prunus persica*, storage conditions, transcriptional regulation

## Abstract

Malate is the predominant organic acid in peach, contributing significantly to fruit sourness and overall organoleptic quality. However, comprehensive understanding of the molecular mechanisms underlying malate accumulation in response to different storage conditions remains limited. In this study, “Hujingmilu” peach was subjected to room temperature (RT, 25°C), low temperature (LT, 4°C), and LT followed by shelf life (LT‐SL, 25°C) treatments to investigate transcriptional regulation mechanisms underlying malate accumulation, with emphasis on biosynthesis, vacuolar storage, and transcription factor regulation. Results demonstrated that LT storage delayed the decline in fruit firmness and maintained higher malate content compared to RT and LT‐SL. Transcriptomic profiling indicated that the expression patterns of malate biosynthetic genes (*PpPEPC1/2*, *PpNAD‐MDH1/2*, *PpNADP‐ME1*) showed limited alignment with malate accumulation. In contrast, genes implicated in proton pump and malate transporter, such as *PpAtpvA1/2/3/4/5*, *PpVp2*, and *PptDT1*, were significantly upregulated under LT conditions, consistent with the observed malate accumulation. Furthermore, LT storage repressed the malate transcriptional repressor *PpTST1* while inducing the candidate regulatory gene *PpMYB62*. These findings provided a comprehensive molecular framework for understanding malate modulation under varying storage conditions.

## Graphical abstract

This study explored malate‐accumulation mechanisms in “Hujingmilu” peaches under different storage conditions. Low temperature (LT) delayed firmness decline and maintained higher malate. Transcriptomics showed malate biosynthetic genes weakly correlate with malate levels, while proton pump and transporter genes were up‐regulated under LT. LT also affects transcription factors (TFs) such as *PpTST1* and *PpMYB62*. Findings indicated that the vacuolar storage and transport system, along with specific TFs, plays crucial roles in the cold‐induced malate accumulation.

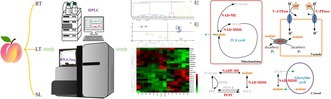

## 1 Introduction

Peach ( *Prunus persica* (L.) Batsch) belongs to the Rosaceae family, is one of the most economically important fruit crops in temperate regions, especially in China, which has the largest cultivation area and yield of peach fruits in the world (FAOSTAT 2025, http://www.fao.org/faostat/en/#home). Peach is favored by consumers worldwide for its rich nutrients (vitamins, sugars, acids, phenolic acids, and flavonoids, etc.) and unique flavor. Flavor affects consumer judgment regarding the overall quality of fruits, is perceived by the sense of taste, oral texture, and smell (Cirilli et al. 2016; Monti et al. 2016). Fruit taste is mainly affected by sourness and sweetness, which is determined by both the content and composition of sugars and organic acids. Malate and citrate are the predominant organic acids found in most ripe fruits, with malate constituting the predominant acid in peach fruit (Baccichet et al. 2021). Except for contribution to fruit taste, organic acids also perform a variety of physiological functions.

Malate serves a crucial role during the initial fruit development stages, whose accumulation has a direct correlation with the provision of substrates necessary for respiratory processes (Igamberdiev and Eprintsev 2016). Malate is involved in the tricarboxylic acid (TCA) and glyoxylate cycles and is additionally synthesized and degraded through glycolytic and gluconeogenic pathways (Wang, Cao, et al. 2023). These complex metabolic processes are regulated by various metabolic enzymes, including phosphoenolpyruvate carboxylase (PEPC), malate dehydrogenase (NAD‐MDH), and malic enzyme (ME) (Etienne et al. 2013).

The majority of malate in fruit is localized within the vacuole (Moskowitz and Hrazdina 1981; Yamaki 1984), which constitutes approximately 90% of most mature fruit cells (Etxeberria et al. 2012; Fontes et al. 2011). Apart from metabolism, the vacuolar storage also affects the final accumulation of organic acids in fruit cells and even plays a dominant role (Etienne et al. 2013). The vacuolar storage of malate in fruit cells depends on a series of transporters and proton pumps, such as aluminum‐activated malate transporters (ALMTs), tonoplast dicarboxylate transporters (tDT), vacuolar H^+^‐ATPases (V‐ATPases), and H^+^‐pyrophosphatases (V‐PPases) proton pumps (Hu et al. 2017; Hurth et al. 2005). Previous research has demonstrated that vacuolar proton pumps and transporters play key roles in the determination of fruit malate. *MdMYB73* was reported to influence malate accumulation by transcriptionally activating the expression of *MdALMT9*, *MdVHA‐A*, and *MdVHP* (Hu et al. 2017). In Arabidopsis, the absence of *AttDT* in mutants displayed significantly reduced levels of malate in leaf tissue, suggesting a functional link between *AttDT* activity and malate content (Medeiros et al. 2017).

Peach is a climacteric fruit whose ripening is regulated by ethylene. After harvest, peach is prone to quick spoilage and tends to soften within 2–3 days at room temperature (Xi et al. 2012). During ripening, with the release of ethylene, changes in the chemical composition and the physical characteristics of the fruit take place, leading to notable modifications in fruit texture, pigmentation, aroma and taste (Paul et al. 2012). Cold storage is extensively utilized to prolong the postharvest shelf life and maintain the quality of fleshy fruits. Peach, categorized as a cold‐sensitive subtropical fruit, is prone to physiological disorders and quality deterioration during long‐term cold storage, a condition referred to as chilling injury (CI), and this phenomenon becomes more pronounced when the fruit is subsequently exposed to warmer temperatures during its shelf life (Roberts et al. 2018; Sevillano et al. 2010). In the present study, postharvest peach was stored at 4°C for 3 weeks; fruits were taken out every week of storage and placed at 25°C for 3 days to simulate shelf life. By comparison with fruit stored at RT, the response of malate accumulation under cold stress was investigated.

## 2 Materials and Methods

### 2.1 Plant Material and Sampling

Peaches ( *Prunus persica* L. Batsch cv. Hujingmilu) were collected from an orchard in Zhangjiagang, Jiangsu Province, China. After harvest, fruits were instantly transported to the laboratory, then fruits with uniform size and without visible defects were selected as experimental material. The selected fruits were randomly divided into two batches; one batch was stored at 25°C for 7 days, named RT group, and sampled at day 0, 1, 3, 5, and 7. Another batch was stored at 4°C for 3 weeks, named LT group, and sampled at day 0, 1, 3, 5, 7, 14, and 21. During LT storage, fruits were taken out every week of storage and placed at 25°C for 3 days as shelf life (SL), named LT‐SL group. Samples were taken on the third day of SL and designated LT7‐SL3, LT14‐SL3, and LT21‐SL3.

At each sampling point, firmness and internal browning (IB) index were determined, then fruit mesocarp was frozen in liquid nitrogen and placed in a −80°C refrigerator for further analysis. For each sampling point, three biological replicates were adopted.

### 2.2 Measurement of Fruit Firmness and IB Index

Fruit firmness was assessed using a hand‐held fruit hardness tester (GY‐3, Zhejiang Top Cloud‐Agri Tech, China) equipped with an 8.0‐mm diameter cylindrical probe. Measurements were conducted to a penetration depth of 10 mm on opposite sides of the equator by removing 1 mm‐thick peel. The results were expressed as kg/cm^2^.

IB is a common symptom of CI observed in peach fruit. IB index was utilized to assess the level of CI, which quantifies the severity of browning by evaluating the extent of discoloration in each fruit after cutting along their axial diameters. The method for measuring the IB index follows the protocol established by Xi et al. (2012), with the grading system defined as follows: Grade 0 indicates no browning; Grade 1 corresponds to < 25% browning; Grade 2 reflects 25%–50% browning; Grade 3 denotes 50%–75% browning; and Grade 4 signifies more than 75% browning. The overall severity of CI was determined using the following formula: 100% × Σ[(internal browning grade) × (number of fruit with that internal browning grade)]/[4 × total number of fruit in each sample].

### 2.3 Determination of Malic and Citric Acids

The extraction of malic and citric acids was performed according to a method described by Ma et al. (2015). Frozen peach pulp was ground to powder in liquid nitrogen, 0.5 g of pulp powder was taken and mixed with 6 mL of distilled water, followed by ultrasonic extraction for 15 min, then the mixture was centrifuged at 5000 *g* for 15 min. The supernatant was taken out and passed through a 0.22 μm aqueous filter membrane for further analysis.

The content of malic and citric acids was determined using an Agilent 1260 Infinity HPLC system equipped with an Athena C18‐WP column (CNW, 4.6 × 250 mm, 5 μm) and a UV detector. The mobile phase was 0.02 mol/L KH_2_PO_4_ (pH 2.4) at a flow rate of 0.8 mL/min. The column temperature was 40°C, and the injection volume was 20 μL. The absorbance value of samples at 210 nm was determined, and the quantitative analysis of malic and citric acids was carried out by comparison with the standard curve. The results were expressed in mg/g fresh weight (Fw).

### 2.4 Transcriptome Sequencing and Data Analysis

Frozen tissue powder of 14 samples with three replicates each was sent to BGI Tech Co. Ltd. (Shenzhen, China) to conduct transcriptome sequencing. Fruit RNA was extracted using the modified CTAB (Bio Basic, Toronto, Canada) method, total RNA was qualified and quantified using the Agilent 2100 Bioanalyzer (Agilent, CA, USA) and the fluorescent dye method. After procedures of sample testing, mRNA isolation, mRNA fragmentation, cDNA synthesis, end repair, adding A and adaptor ligation, PCR, library testing, and circularization, DNA molecules were sequenced at a DNBSEQ platform.

After sequencing, the reads with low quality, junction contamination, and high content of unknown base N were filtered. Then the clean reads were aligned to the reference genome (GDR: https://www.rosaceae.org/species/prunus_persica/genome_v2.0.a1). Expression level of the gene was calculated by RSEM (v1.3.1) (Li and Dewey 2011) and expressed as FPKM (fragments per kilobase of exon model per million mapped fragments of each gene).

### 2.5 Real‐Time Quantitative PCR (RT‐qPCR) Validation

Total RNA was isolated from frozen flesh tissue using the protocol provided by the MiniBEST Plant RNA Extraction Kit (TaKaRa, Japan). First‐strand cDNA synthesis was performed according to the manufacturer's instructions using the PrimeScript RT Master Mix (TaKaRa, Japan). For RT‐qPCR analysis, the Applied Biosystems QuantStudio 5 Real‐Time PCR System (Applied Biosystems, USA) was utilized with ChamQ Universal SYBR qPCR Master Mix (Vazyme, China). The cycling protocol included an initial denaturation step at 95°C for 30 s, followed by 40 cycles of denaturation at 95°C for 10 s and annealing at 60°C for 30 s. Nine genes were randomly chosen for RT‐qPCR, which were listed in Table S1. Translation elongation factor 2 (TEF 2) was selected as an internal control to normalize sample differences (Tong et al. 2009), and the value of cycle threshold (*C*_t_) was obtained for both the reference and target genes. The relative gene expression was calculated using the “Comparative $2^{-∆∆C_{T}}$” method (Livak and Schmittgen 2001). RNA isolation and cDNA syntheses were performed in triplicate.

### 2.6 Statistical Analysis

All data were firstly processed using Microsoft Word 2019, with results presented as means ± standard deviation (SD). Subsequently, significant difference between samples was revealed by the analysis of variance followed by Duncan's multiple range test (*p* < 0.05) utilizing SPSS 18.0 software (SPSS Inc., Chicago, IL, USA). The figures included in this study were generated using Origin Pro 2022 (OriginLab, Northampton, MA, USA).

## 3 Results

### 3.1 The Effect of Storage Temperature on Fruit Firmness and IB Index

**FIGURE 1:**
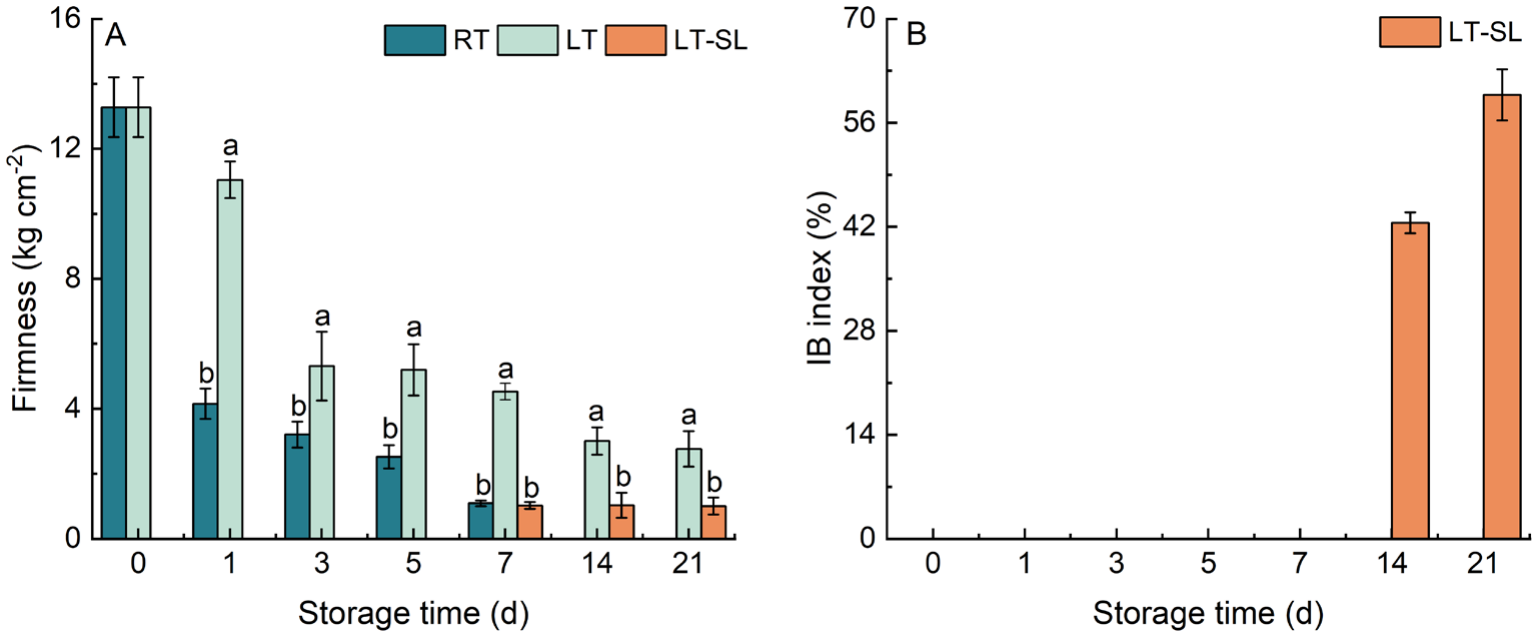
The effect of storage temperature on fruit firmness (A) and IB index (B). Blank bars at the sampling point in Figure (B) means no chilling injury in corresponding samples. RT indicates storage at room temperature (25°C) and LT indicates storage at low temperature (4°C); LT‐SL at days 7, 14, and 21 refers to samples collected on the third day of shelf life following 7, 14, and 21 days of LT storage. The data are presented as means ± standard deviation (SD) from three independent replicates. Significant differences among groups, denoted by different letters, were identified using Duncan's test at a significance level of 0.05, while the same letter means no significant difference between groups.

Fruit firmness tended to decrease whether stored at LT or RT (Figure 1A). Peach firmness dropped dramatically from 13 to 4 kg/cm^2^ after 1 day of storage at 25°C. Low temperature significantly inhibited the decrease in fruit firmness by contrast with RT storage. At LT‐SL stage, fruit firmness observably reduced.

During 3 weeks of LT storage, fruits did not develop any IB symptoms. IB appeared after shelf life following 14 and 21 days of LT, with greater severity in LT21‐SL3 (Figure 1B).

### 3.2 The Effect of Storage Temperature on Fruit Organic Acids

**FIGURE 2:**
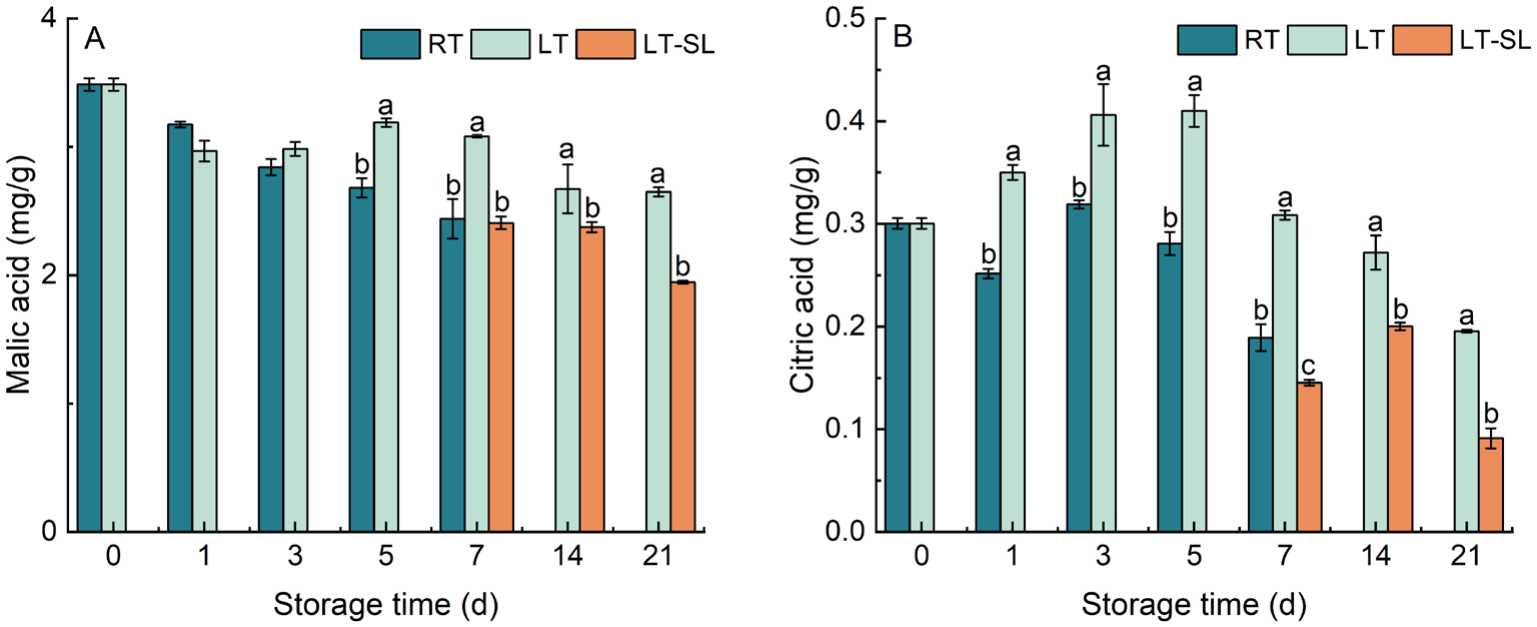
The effect of storage temperature on the content of malic acid (A) and citric acid (B). RT indicates storage at room temperature (25°C) and LT indicates storage at low temperature (4°C), LT‐SL at days 7, 14, and 21 refers to samples collected on the third day of shelf life following 7, 14, and 21 days of LT storage. The data are presented as means ± standard deviation (SD) from three independent replicates. Significant differences among groups, denoted by different letters, were identified using Duncan's test at a significance level of 0.05, while the same letter means no significant difference between groups.

As shown in Figure 2, the malate content of “Hujingmilu” peaches is about 3.5 mg/g at harvest. A successive decrease was observed in malate during RT storage (Figure 2A). The malate content of peach fruits stored at 4°C was significantly higher than that at 25°C on days 5 and 7. When peaches were moved to SL, the level of malate decreased.

Citrate content was generally reduced during RT storage, specifically, decreased slightly after 1 day of 25°C storage, then increased above the level at harvest, finally decreased after the third day (Figure 2B). By contrast, LT significantly stimulated citrate synthesis in peach fruits. The content of citrate showed a clear trend of first increasing and then decreasing under LT storage, with a peak value of 0.4 mg g^−1^ observed on day 5. A significant decrease in citrate content was observed at LT‐SL stage.

### 3.3 Transcriptomic Analysis

**FIGURE 3:**
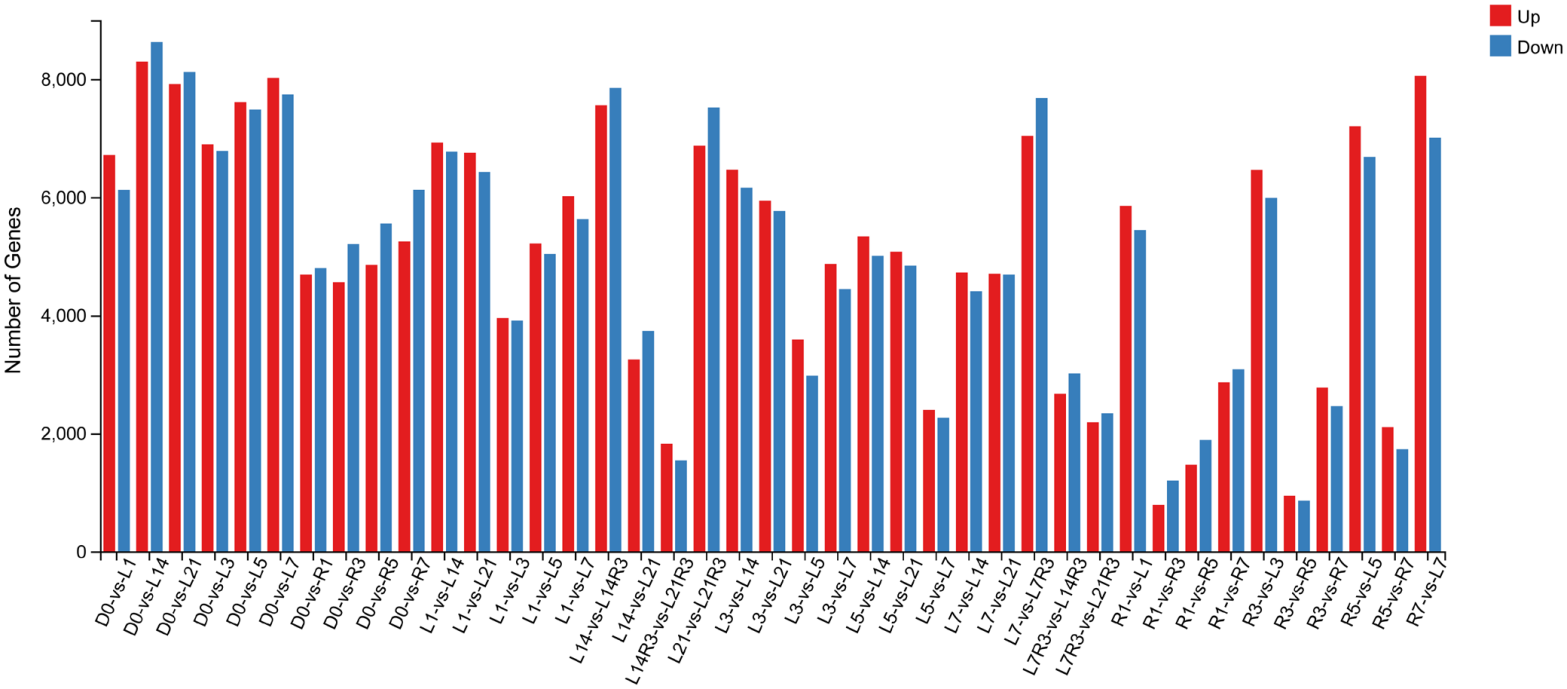
Number of DEGs between the pairwise sample. Up: up‐regulated genes; Down: down‐regulated genes. L: LT storage, R: RT storage. Number after L/R: storage days.

A total of 42 samples composed of three independent biological replicates of each sampling point were measured in this project using the DNBSEQ platform, yielding an average of 6.48 Gb of clean bases per sample (Table S2). The average mapping ratio of clean reads to reference genome was 95.5%, ranging from 89.6% to 98.5% (Table S3). Differential expression analysis was performed to discover differential expression genes (DEGs) using the DESeq2 (v1.4.5) with adjusted *p*‐value ≤ 0.05 (Love et al. 2014). To explore the regulation of different storage conditions on DEGs, the numbers of up‐regulated and down‐regulated DEGs between two groups were presented in Figure 3.

To evaluate the reliability of the RNA‐Seq data, RT‐qPCR was performed on nine randomly selected genes representing a range of expression levels and functional categories. As shown in Figure S1, the expression levels obtained by RT‐qPCR were highly consistent with those from RNA‐Seq, with the average *R* ^2^ > 0.87.

**FIGURE 4:**
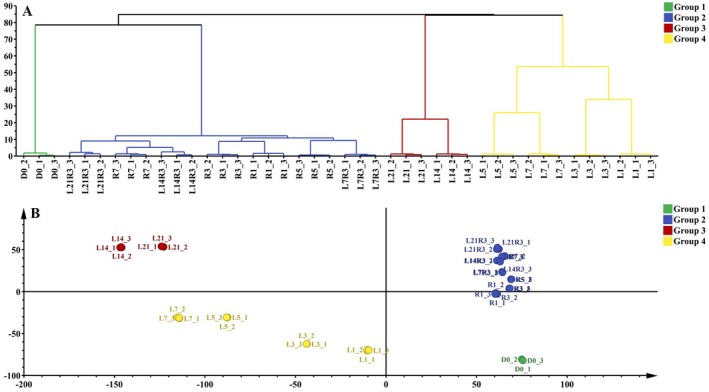
HAC (A) and PCA (B) of total genes expression for 42 samples. D: storage days. L: LT storage, R: RT storage. Number after L/R: storage days. 1/2/3: three replicates.

To obtain a comprehensive overview of the RNA‐Seq data, hierarchical clustering analysis (HCA) and principal component analysis (PCA) were performed. The dendrogram generated by HCA, with branches distinguished by different colors, revealed four major clusters among the samples, forming two monophyletic branches corresponding to RT and LT storage (Figure 4A). The D0 samples clustered distinctly, constituting Group 1. Group 2 comprised all RT samples, including both normal RT and LT‐SL samples. These two groups clustered together to form a large monophyletic “RT” branch. Group 3 included LT14 and LT21 samples, which formed into another monophyletic branch with Group 4 that consisted of LT1, LT3, LT5, and LT7. The PCA results corroborated the clustering patterns observed in the HCA, demonstrating consistent sample separation (Figure 4B).

### 3.4 Genes Expression Associated With Malate Accumulation

#### 3.4.1 The Expression of Genes Related to Malate Biosynthetic Pathway

**FIGURE 5:**
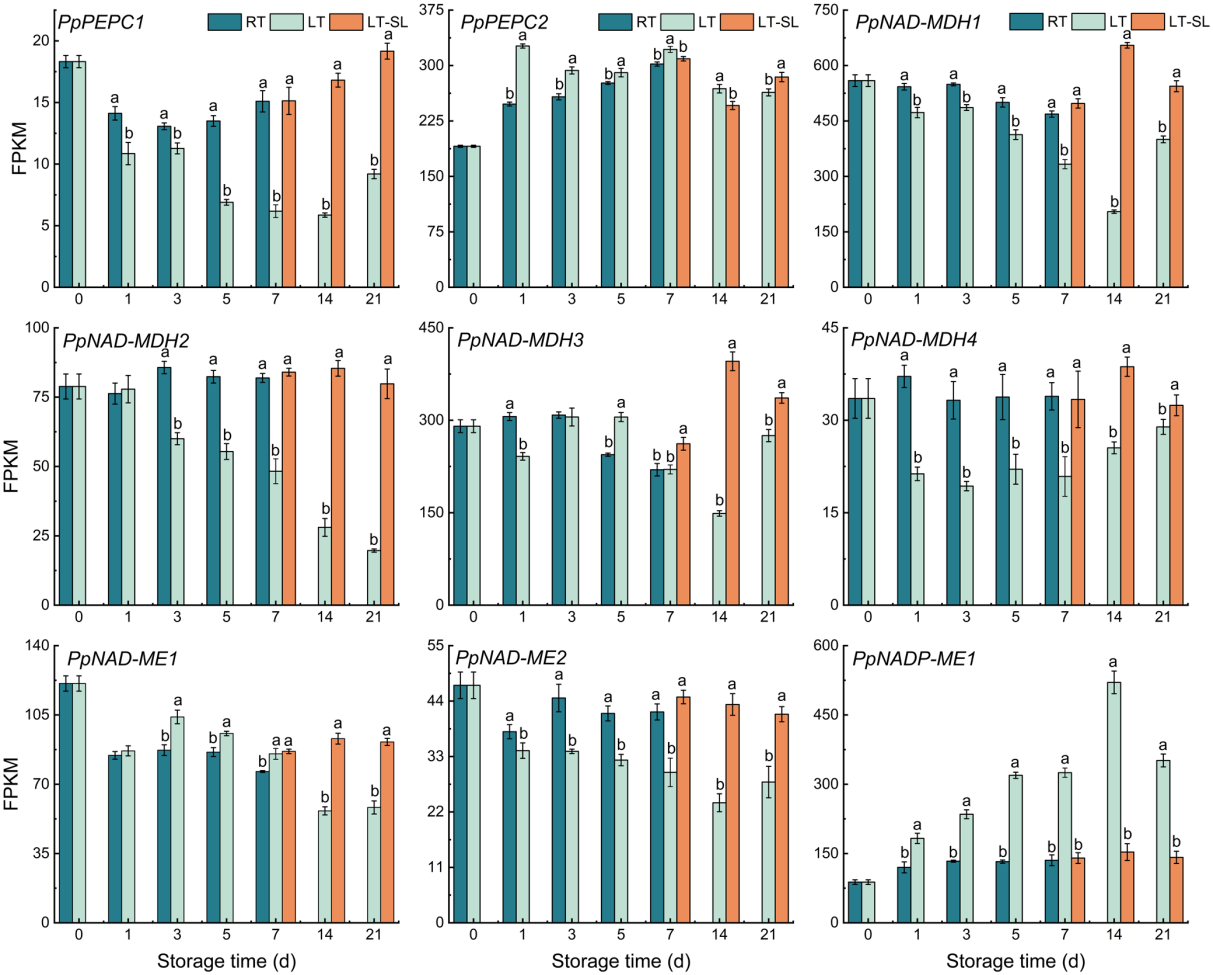
The effect of storage temperature on the expression of genes related to malate biosynthetic pathway. RT indicates storage at room temperature (25°C) and LT indicates storage at low temperature (4°C), LT‐SL at days 7, 14, and 21 refers to samples collected on the third day of shelf life following 7, 14, and 21 days of LT storage. The data are presented as means±standard deviation (SD) from three independent replicates. Significant differences among groups, denoted by different letters, were identified using Duncan's test at a significance level of 0.05, while the same letter means no significant difference between groups.

The gene IDs from GDR (https://www.rosaceae.org/) and their corresponding names used in this study are listed in Table S4. In general, the transcript levels of *PpPEPC1* showed a downward trend during the first 2 weeks of LT storage, while was remarkably elevated at the LT‐SL stage (Figure 5). Unlike *PpPEPC1*, the expression of *PpPEPC2* increased during the first week of storage, with a higher level found in LT storage. Specifically, the expression levels on day LT7‐SL3 and LT14‐SL3 were marginally reduced compared to LT. However, an elevated abundance of *PpPEPC2* was observed on day LT21‐SL3.

*PpNAD‐MDH1* of RT and LT both showed a downward trend within the first 7 days of storage, while LT led to a more obvious decline (Figure 5). The expression level of *PpNAD‐MDH1* was significantly elevated when transferring to SL. The expression pattern of *PpNAD‐MDH3* was generally similar to *PpNAD‐MDH1* with the exception of day 5, when LT was higher than RT. The transcript abundance of *PpNAD‐MDH2* exhibited a consistent declining trend throughout the entire LT period, while it slightly increased at RT storage. At LT‐SL sampling point, *PpNAD‐MDH2* expressed remarkably higher than LT. The overall expression of *PpNAD‐MDH4* exhibited minimal variation during RT storage, while it was visibly inhibited by LT. A significant increase in *PpNAD‐MDH4* level was observed at the LT‐SL period.

The expression level of *PpNADP‐ME1* was significantly induced by LT, increased continually until the highest point at day 14. Under RT and LT‐SL period, *PpNADP‐ME* level was relatively stable (Figure 5). Compared to day 0, *PpNAD‐ME1* expressed depressedly; RT had little effect on its abundance, while there was a slight induction by LT. At day 3, 5, and 7, *PpNAD‐ME1* under LT storage showed higher expression level. Nevertheless, the performance of *PpNAD‐ME2* in RT and LT storage was inverse. The expression of *PpNAD‐ME1/2* was observably stimulated when transferring to SL for 3 days.

#### 3.4.2 The Expression of Genes Related to Malate Vacuolar Storage

**FIGURE 6:**
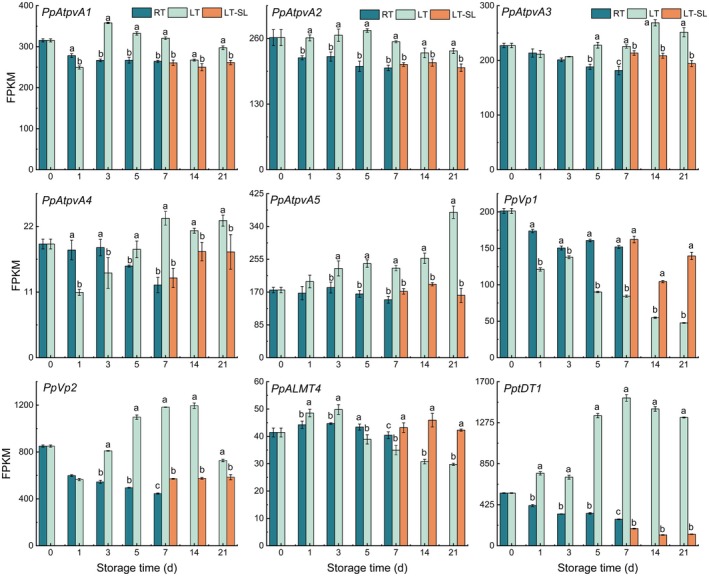
The effect of storage temperature on the expression of genes related to malate vacuolar storage. RT indicates storage at room temperature (25°C) and LT indicates storage at low temperature (4°C), LT‐SL at days 7, 14, and 21 refers to samples collected on the third day of shelf life following 7, 14, and 21 days of LT storage. The data are presented as means ± standard deviation (SD) from three independent replicates. Significant differences among groups, denoted by different letters, were identified using Duncan's test at a significance level of 0.05, while the same letter means no significant difference between groups.

As shown in Figure 6, the mRNA abundance of *PpAtpvA1* and *PpAtpvA2* showed a similar change trend, was induced by LT, while remained basically unchanged during RT and LT‐SL stage. During RT storage, there was a gradually declining trend in *PpAtpvA3* and *PpAtpvA4* expression during the first 7 days. However, under LT storage, the expression level of *PpAtpvA3/4* exhibited an initial transient decrease followed by a pronounced increase, which was significantly higher than that observed in the RT and LT‐SL group. During RT storage, the expression of *PpAtpvA5* remained stable; however, under LT conditions, its expression level exhibited a sustained increase and was 2.33 times that of LT‐SL on the 21st day. *PpVp1* and *PpVp2* exhibited a similar expression mode during RT and LT‐SL storage, while in LT, the two genes showed completely opposite change trends (Figure 6). The transcriptional level of *PpVp1* progressively decreased with extended refrigeration time, was 52%, 52%, and 34% of the levels observed at shelf life after 1, 2, and 3 weeks of low‐temperature storage, respectively. The expression level of *PpVp2* continuously increased, reaching the peak at day 14 of LT storage, which is twice as high as the LT‐SL, followed by a decline on day 21.

The mRNA abundance of two ALMT genes (Prupe.5G220200 and Prupe.5G110600) was extremely low with FPKM < 1, which was not shown in Figure 6. LT significantly increased the mRNA level of *PpALMT4* at day 1 and 3, but downregulated its expression at the rest of the sampling points. One tDT gene—PptDT1 was considered in this study. LT significantly stimulated the expression of *PptDT1* during the entire storage period, while under RT stage, its abundance kept decreasing. On the seventh day of LT, the FPKM value of *PptDT1* reached the peak, being 1.8‐fold higher than the level on day 0 (Figure 6).

#### 3.4.3 The Expression of Transcription Factor (TFs) Related to Malate Accumulation

**FIGURE 7:**
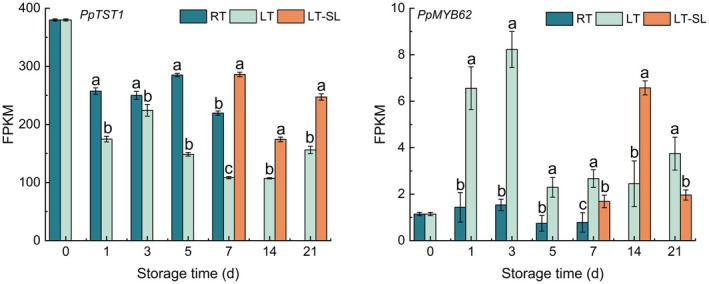
The effect of storage temperature on the expression of TFs related to malate vacuolar storage. RT indicates storage at room temperature (25°C) and LT indicates storage at low temperature (4°C), LT‐SL at days 7, 14, and 21 refers to samples collected on the third day of shelf life following 7, 14, and 21 days of LT storage. The data are presented as means ± standard deviation (SD) from three independent replicates. Significant differences among groups, denoted by different letters, were identified using Duncan's test at a significance level of 0.05, while the same letter means no significant difference between groups.

In general, the expression level of *PpTST1* during storage was lower than day 0 (Figure 7). By contrast, LT markedly reduced the abundance of *PpTST1* compared to RT and LT‐SL stage. During RT storage, the mRNA level of *PpMYB62* showed minimal change. However, its expression was significantly provoked by LT at day 1 and 3, followed by a sharp decrease (Figure 7). Except for day 14, *PpMYB62* in LT was markedly higher than LT and LT‐SL.

## 4 Discussion

In this study, storage temperatures significantly impacted fruit firmness, IB index, and the metabolism of malate and citrate. “Hujingmilu” peach is classified as melting flesh, whose ripening process is accompanied by rapid firmness loss (Veerappan et al. 2021). LT is widely utilized as a postharvest strategy to retard ripening processes and enhance storage stability, particularly in climacteric fruits, such as tomato, peach, and apricot (Tucker et al. 2017). LT delayed firmness decline, whereas a dramatic decrease in fruit firmness was observed upon transfer to the SL stage (Figure 1A), indicating that LT inhibited the ripening of peach fruit. Liu et al. (2024) found that cold exposure activates integrated IAA biosynthesis in peach fruit; however, post‐cold recovery at room temperature significantly diminished cold stress resistance, as evidenced by reduced active IAA levels. This temperature‐dependent susceptibility aligns with observations in other crops, where cold‐induced tolerance is transient and rapidly reversed upon return to ambient conditions (Jankauskiene et al. 2022; Lafuente and Romero 2022; Wang, Yu, et al. 2023). In the present study, IB symptoms appeared only in the SL stage after 14 days of LT storage, suggesting that exposure to RT diminished cold tolerance in peaches, resulting in the manifestation of IB, which was also consistent with our previous findings (Cai et al. 2020).

Organic acids play a crucial role as carbon sources and flavor compounds in fruit. In ripe peach, malate and citrate are primarily the dominant organic acids, similar to what is observed in many other fleshy fruits (Jiang et al. 2023). Organic acids accumulate at fruit development stage, while a decline in levels occurs in most fleshy fruits during the ripening process, which are used as respiratory substrates (Etienne et al. 2002; Giovannoni et al. 2017). Similar results were also found in our study that malate consistently decreased, and citrate also showed a downward trend overall during RT storage (Figure 2A). During LT storage, malate and citrate maintained a higher level, suggesting that LT delayed the degradation of organic acids, which may be due to the inhibiting effect of LT on fruit ripening. When fruit was transferred from LT to SL, malate and citrate significantly declined, accompanied by severe IB symptoms (Figures 1B and 2), indicating that CI is associated with organic acid metabolism. In peach fruit, Zhou et al. (2021) also proposed that hot air suppressed CI by regulating organic acid metabolism. Similar results were also found in apples that flesh browning tissues caused by CI exhibited significantly reduced levels of organic acids, which may relate to a higher proportion of lactic acid/malic acid level and the up‐regulation of lactate dehydrogenase expression (Wang, Li, et al. 2023).

Within fruit cells, malate is the primary form of malic acid, accumulated via both biosynthetic pathways and subsequent storage in the vacuoles (Chi et al. 2025). The metabolism of malate and citrate within the fruit itself is the primary factor contributing to variations in the acidity of fleshy fruits (Etienne et al. 2013). In cytosol, PEPC catalyzes the conversion of phosphoenolpyruvate (PEP) to oxaloacetate (OAA), which is subsequently reduced to malate by the cytosolic NAD‐dependent MDH (NAD‐cytMDH) (Etienne et al. 2013). In this study, it was observed that the trend of malate levels exhibited a tendency to be inversely associated with the expression pattern of *PpPEPC1*, and also showed no significant correlation with the expression of the gene *PpPEPC2* (Figures 2 and 5). Previous studies found that PEPC does not contribute to the differences in malate content observed between peach cultivars with low and high acidity (Moing et al. 2010). Besides, the observed increase in PEPC activity, coupled with unchanged malate levels, suggests that this enzyme may facilitate a glycolytic bypass for PK (Borsani et al. 2009).

NAD‐cytMDH mainly catalyzes the synthesis of malate from OAA in reversible conversion, which was responsible for malate synthesis in fruit (Etienne et al. 2013). However, the expression of two cytosol NAD‐MDH genes (*PpNAD‐MDH1/2*) in our study showed an almost opposite trend with malate level (Figures 2 and 5). *PpNAD‐MDH1* was reported to have no impact on fruit acidity (Etienne et al. 2002). Sequence blast at mRNA level revealed that *PpNAD‐MDH2* shared 77.18% identity with *PpNAD‐MDH1*, explaining the similarity of their transcript abundance. In fruit mitochondria, malate is oxidized predominantly to OAA via NAD‐dependent MDH (NAD‐mtMDH) or converted to pyruvate by NAD‐dependent ME (NAD‐mtME), which feeds or disrupts the citric acid cycle (Etienne et al. 2013; Sweetman et al. 2009). In cytosol, NADP‐ME catalyzed the most likely direction of malate to pyruvate, involving the decrease in malate content (Etienne et al. 2013). At LT‐SL stage, one mitochondrial NAD‐mtMDH gene (*PpNAD‐MDH3*) showed elevated levels than LT, suggesting that SL accelerated the depletion of malate. *PpNAD‐MDH4* is a glyoxysomal NAD‐MDH, which mainly catalyzes the direction of malate to OAA. Our results showed that LT inhibited *PpNAD‐MDH4* expression compared to RT and LT‐SL stage, indicating that LT suppressed the consumption of malate, which was in consistency with malate level. The expression level of mitochondrial *PpNAD‐ME*2 illustrated higher consumption at warmer temperature storage, which is in accordance with malate change trend. *PpNAD‐ME1* and *PpNADP‐ME1* showed higher expression in LT group, indicating higher consumption in malate during 4°C storage. These findings tentatively suggested that the expression of key enzyme genes involved in malate biosynthesis may not be the sole determinant of malate levels.

Following its synthesis in the cytosol, malate is transported into the vacuoles within fruit cells. Malate accumulation within fruit cells is predominantly regulated through vacuolar storage, while metabolic processes adjust accordingly to maintain cytosolic malate levels (Etienne et al. 2014). Malate uptake into vacuoles is primarily facilitated by ALMT and tDT transporters, whereas proton pumps drive proton translocation into the vacuolar compartment. Ultimately, malate undergoes protonation and accumulates in the vacuoles of fruit cells predominantly as malic acid (Chi et al. 2025). In this study, the expression pattern of *PpAtpvA1/2/3/4/5* and *PpVp2* were elevated by LT, similar to the change of malate, suggesting that the positive role of proton pumps in malate accumulation. In apples, the elevated malate levels observed in fruit stored near freezing temperatures corresponded with increased expression of V‐ATPase and V‐PPase genes (Shu et al. 2024). Among ALMTs family, ALMT9 was the most extensively studied and recognized as a principal regulator of malate transport in *Arabidopsis*, tomato and apple (Huang et al. 2021). During storage, ALMT9 transcripts were almost undetectable in the flesh of “Hujingmilu” peaches, implying that ALMT9 is unlikely to be a key determinant of malate accumulation in this cultivar. Yu et al. (2021) demonstrated that *PpALMT1* promotes malate accumulation during peach fruit development. However, 85% of our samples exhibited FPKM values < 1 (indicating negligible expression). Notably, among ALMT family genes, overexpression of *PpALMT4* increased malate content in yellow‐fleshed peaches (Chen et al. 2025), whereas LT treatment significantly elevated *PpALMT4* expression only at days 1 and 3. This discrepancy may stem from differences in peach cultivars and postharvest processing. Regarding *PptDT1*, its abundance was significantly increased under LT, accompanied by an elevated malate content (Figures 2 and 6), consistent with the study in apple showing that *MdtDT* modulates malate accumulation and vacuolar pH (Hu et al. 2017). consistently correlated with malate content.

Transcriptional control constitutes a critical intrinsic mechanism governing malate accumulation in fruit, with TFs serving as central regulators. In apple, Hu et al. (2017, 2016) revealed that the R2R3‐MYB transcription factors MYB1 and MYB73 modulate malate content and vacuolar pH through the activation of vacuolar transporter genes. While Jia et al. (2021) found that another R2R3‐MYB transcription factor‐MdMYB44 negatively regulates malate accumulation in apple. A MADS‐box TF‐*EjAGL18* was found to negatively regulate malic acid accumulation in loquat by repressing the expression level of *EjtDT1* (Chi et al. 2025). Current research on organic acids in peach has predominantly concentrated on structural genes, while the TFs that were confirmed to influence organic acid accumulation remain poorly understood. In peach, a key gene‐*PpTST1*, encoding a tonoplast sugar transporter, was reported to negatively regulate organic acid accumulation by reducing the expression of genes of organic acid transport (Wang et al. 2022). In the present study, LT inhibited *PpTST1* expression compared with RT and LT‐SL, while LT increased the gene expression of *PptDT1*, *PpAtpvA1*, and *PpVp2* and malate content during storage, which indirectly confirmed the transcriptional inhibitory activity of *PpTST1* on the accumulation of organic acids. Wang, Cao, et al. (2023) reported that MYB62 was a candidate gene that positively correlated with malate content in peaches. Here, LT induced the expression of *PpMYB62* except for day 14, which was in consistence with the effect of LT on malate content (Figure 7).

**FIGURE 8:**
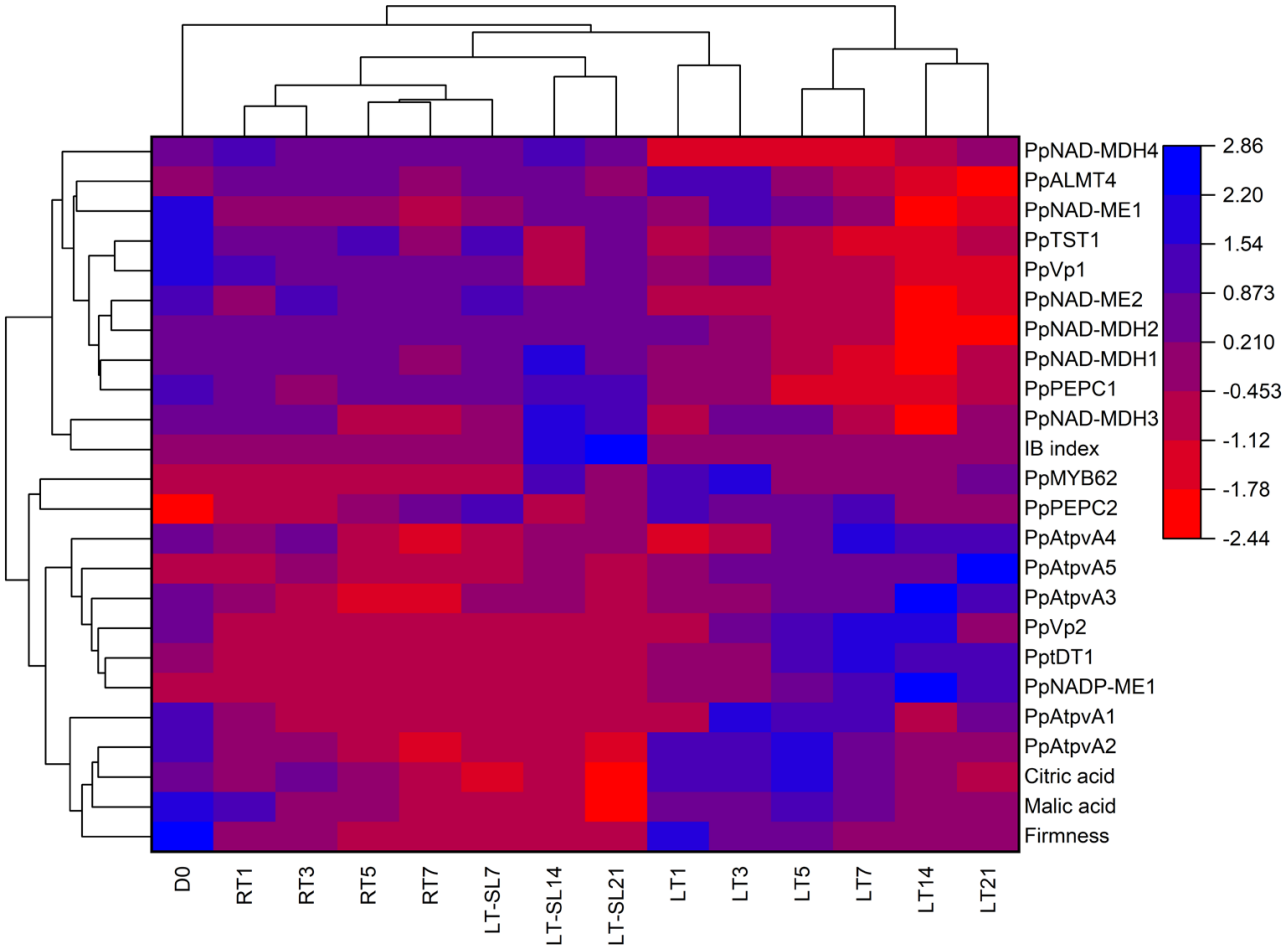
Cluster heatmap analysis of full‐data during storage. RT indicates storage at room temperature (25°C) and LT indicates storage at low temperature (4°C), LT‐SL refers to samples collected on the third day of shelf life following 7, 14, and 21 days of LT storage. Numbers after RT/LT/LT‐SL indicate storage days.

**FIGURE 9:**
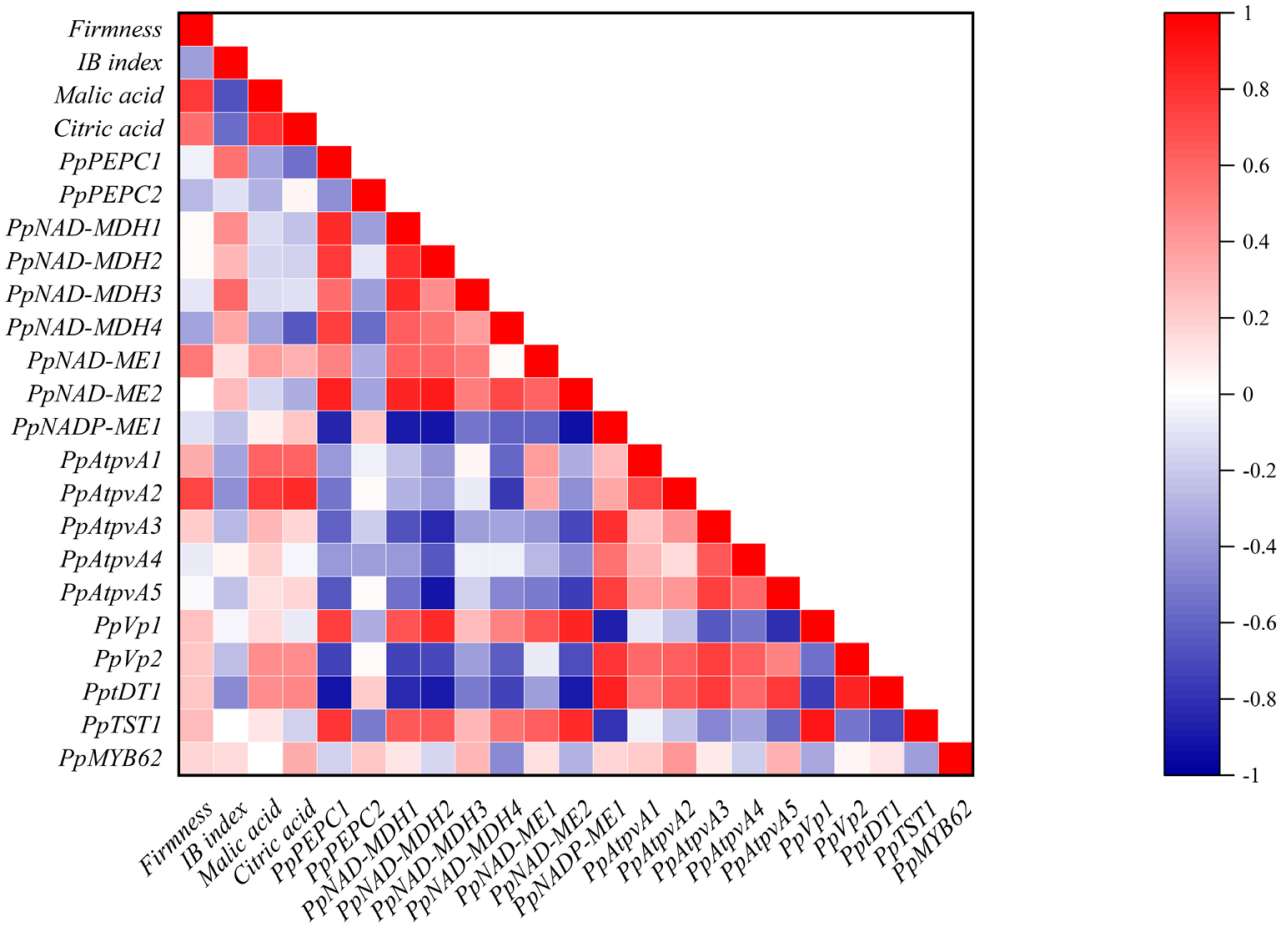
Pearson correlation analysis of full data during RT, LT, and LT‐SL storage.

**FIGURE 10:**
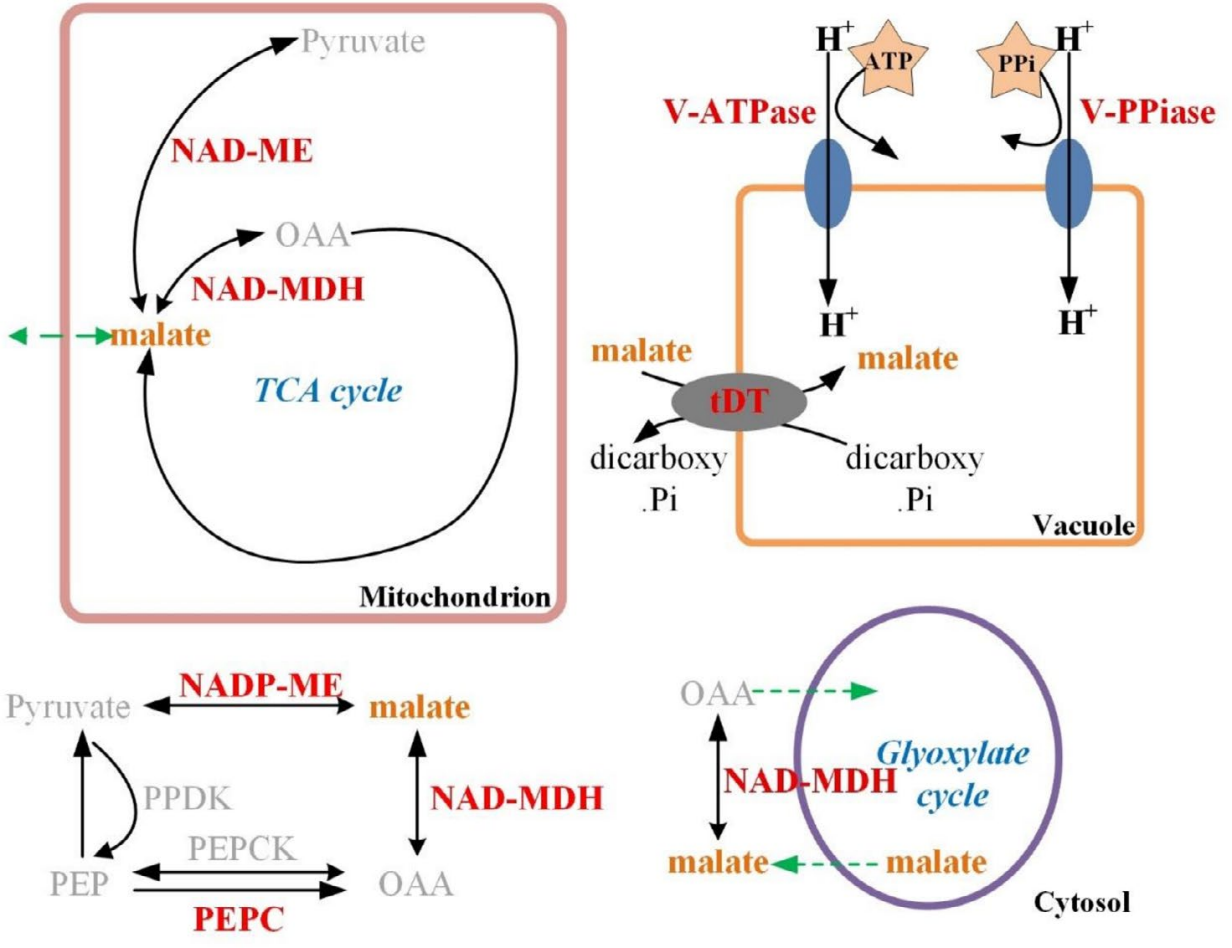
A simplified model illustrating the roles of candidate genes (highlighted in red) identified in peach fruit in the regulation of malate accumulation. The predominant direction of reversible reactions is indicated by the large arrow, while dashed green arrows represent the transport of malate. NAD‐MDH, NAD‐malate dehydrogenase; NAD‐ME, NAD‐malic enzyme; NADP‐ME, NADP‐malic enzyme; OAA, oxaloacetate; PEP, phosphoenolpyruvate; PEPC, phosphoenolpyruvate carboxylase; PEPCK, phosphoenolpyruvate carboxykinase; PPDK, pyruvate orthophosphate dikinase. V‐ATPase, vacuolar H^+^‐ATPase; V‐PPiase, vacuolar H^+^‐pyrophosphatase.

Cluster heatmap analysis was conducted to provide a comprehensive overview of the entire dataset (Figure 8). The analysis revealed two main clusters based on storage duration and temperature conditions. One cluster comprised D0, RT1, RT3, RT5, RT7, and LT‐SL7, LT‐SL14, LT‐SL21, corresponding to samples stored at warm temperatures. The other cluster included all samples stored under low‐temperature conditions from LT1 to LT21, indicating a clear distinction between peaches stored at RT. All indicators considered in this study were clustered into two categories. At Day 0, fruit was characterized by high levels of firmness, malate, and expression of *PpTST1*, *PpVp1*, *PpNAD‐ME1*, and *PpNAD‐ME2*. Throughout storage, LT significantly up‐regulated the expression of *PpMYB62*, *PptDT1*, *PpNADP‐ME1*, *PpVp2*, *PpAtpvA3*, *PpAtpvA5*, *PpAtpvA4*, *PpAtpvA2*, *PpAtpvA1* and maintained higher fruit firmness and organic acids. These findings were further supported by correlation heatmap analysis (Figure 9), which revealed that the LT‐induced increase in malate content was associated with the expression of genes encoding malate transporters, proton pumps, and TFs. A simplified model was proposed according to predecessors' research (Etienne et al. 2013), which illustrated the roles of candidate genes in the regulation of malate accumulation in peach (Figure 10).

## 5 Conclusion

The present study indicates that LT storage better preserves fruit firmness compared to RT and LT‐SL conditions. CI symptoms were observed only when peaches were transferred to shelf life after 2 or 3 weeks of LT storage. Notably, LT storage delayed the depletion of malate and citrate relative to RT and LT‐SL treatments. Transcriptome analysis suggests that the genes associated with malate biosynthesis might not directly dictate the final malate content. Instead, the elevated malate levels observed under LT conditions correlated with the upregulation of genes involved in proton pump, malate transporters, and their related transcription factors. These results together indicated that under different postharvest storage conditions, malate in peach was predominantly controlled by vacuolar storage rather than biosynthesis.

## Author Contributions

**Xu Wang:** investigation, methodology. **Zhuyi Gao:** investigation. **Hongfang Cai:** conceptualization, supervision, investigation, writing – original draft. **Zheng Zhang:** investigation. **Zewen Xiong:** investigation. **Shuai Han:** data curation, formal analysis, writing – review and editing. **Jiqing Shi:** resources, project administration.

## Conflicts of Interest

The authors declare no conflicts of interest.

## Supporting information


**Table S1:** Primers for real‐time quantitative PCR used for validation of RNA‐Seq results.
**Table S2:** Clean reads quality metrics for 42 RNA‐Seq samples.
**Table S3:** Summary of genome mapping for RNA‐Seq samples.
**Table S4:** Gene ID in GDR (https://www.rosaceae.org/) and corresponding gene name in article.
**Figure S1:** The nine expression genes randomly selected were validated through real‐time quantitative PCR to assess RNA‐Seq data. This comparison relies on relative expression data obtained from real‐time quantitative PCR and FPKM values derived from the RNA‐Seq results. The line represents the orthogonal fit to the data, with the correlation coefficient (*R* ^2^) displayed. FPKM refers to fragments per kilobase per million mapped fragments.

## Data Availability

The data that support the findings of this study are available from the corresponding author upon reasonable request.
