# Supplementary material for: Comprehensive Analysis of Malate Accumulation in Peaches in Response to Cold Stress Based on Transcriptomics
Source: Food Sci Nutr. 2026 Jan 7;14(1):e71425. doi: 10.1002/fsn3.71425 (PMC12778420; doi:10.1002/fsn3.71425)
Supplement: Supplementary file 1 — Table S1: Primers for real‐time quantitative PCR used for validation of RNA‐Seq results. Table S2: Clean reads quality metrics for 42 RNA‐Seq samples. Table S3: Summary of genome mapping for RNA‐Seq samples. Table S4: Gene ID in GDR (https://www.rosaceae.org/) and corresponding gene name in article. Figure S1: The nine expression genes randomly selected were validated through real‐time quantitative PCR to assess RNA‐Seq data. This comparison relies on relative expression data obtained from real‐time quantitative PCR and FPKM values derived from the RNA‐Seq results. The line represents the orthogonal fit to the data, with the correlation coefficient (R 2) displayed. FPKM refers to fragments per kilobase per million mapped fragments. [file FSN3-14-e71425-s001.docx]

**Table S1.** Primers for real-time quantitative PCR used for validation of RNA-Seq results.

| Gene name | Forward primer(5′-3′) | Reverse primer(5′-3′) |
| --- | --- | --- |
| *PpTEF2* | GGTGTGACGATGAAGAGTGATG | TGAAGGAGAGGGAAGGTGAAAG |
| *PpADH1* | AACGCCCGACTAGTTTGTTG | CGATCATTCTTCGGCAAATC |
| *PpAAT1* | TTGGAGAGGTTTGAGGAGGA | AGCCCACACAACACAAGACA |
| *PpSPS1* | AAGGCTTGCTTGGTGGGTTA | TGGGACTGTCATTTGGGAAT |
| *PpSS1* | TCGCCACCTTTCTTCTGTTA | CTCGAAACCCATTCCTTGTA |
| *PpNI1* | AGTTCAGGAGAGAGTTGATGTG | CTACCAGACGGTTGTTGAGT |
| *PpLOX1* | TCACTACGACAAGCGGAACG | GGTAGGACGGTTTGGCACAT |
| *PpFAD1* | CGCTCCTTCTCCTATGTCTTTT | TGGAGGATTAGACCGACTGTG |
| *PpME1* | TACGCATCCACACGGTCTTT | CCATCCCATGACAGCCAAGA |
| *PpNA1* | TCCGAGGGCAGAGCGAAGAAC | TTGTGGAGGCTTGTGAGGATTGG |

**Table S2.** Clean reads quality metrics for 42 RNA-Seq samples.

| Sample | Total Raw Reads (Mb) | Total Clean Reads (Mb) | Total Clean Bases (Gb) | Clean Reads Q20  (%) | Clean Reads Q30  (%) | Clean Reads Ratio  (%) |
| --- | --- | --- | --- | --- | --- | --- |
| D0_1 | 43.69 | 43.24 | 6.49 | 99.28 | 96.18 | 98.97 |
| D0_2 | 43.69 | 43.14 | 6.47 | 99.3 | 96.31 | 98.75 |
| D0_3 | 43.69 | 43.25 | 6.49 | 99.21 | 95.88 | 99 |
| R1_1 | 43.69 | 43.27 | 6.49 | 99.28 | 96.24 | 99.03 |
| R1_2 | 43.69 | 43.14 | 6.47 | 99.27 | 96.13 | 98.75 |
| R1_3 | 43.69 | 43.19 | 6.48 | 99.27 | 96.16 | 98.86 |
| R3_1 | 43.69 | 43.19 | 6.48 | 99.23 | 95.96 | 98.85 |
| R3_2 | 43.69 | 43.19 | 6.48 | 99.26 | 96.12 | 98.85 |
| R3_3 | 43.69 | 43.2 | 6.48 | 99.2 | 95.8 | 98.87 |
| R5_1 | 43.69 | 43.19 | 6.48 | 99.27 | 96.22 | 98.86 |
| R5_2 | 43.69 | 43.27 | 6.49 | 99.23 | 96.02 | 99.04 |
| R5_3 | 43.69 | 43.24 | 6.49 | 99.23 | 95.95 | 98.96 |
| R7_1 | 43.69 | 43.23 | 6.48 | 99.27 | 96.11 | 98.95 |
| R7_2 | 43.69 | 43.15 | 6.47 | 99.14 | 95.65 | 98.77 |
| R7_3 | 43.69 | 43.08 | 6.46 | 99.31 | 96.31 | 98.6 |
| L1_1 | 43.69 | 43.26 | 6.49 | 99.24 | 96 | 99.01 |
| L1_2 | 43.69 | 43.23 | 6.49 | 99.25 | 96.09 | 98.95 |
| L1_3 | 43.69 | 43.24 | 6.49 | 99.26 | 96.09 | 98.97 |
| L3_1 | 43.69 | 43.23 | 6.48 | 99.28 | 96.13 | 98.94 |
| L3_2 | 43.69 | 43.27 | 6.49 | 99.23 | 96 | 99.05 |
| L3_3 | 43.69 | 43.27 | 6.49 | 99.22 | 95.88 | 99.04 |
| L5_1 | 43.69 | 43.27 | 6.49 | 99.16 | 95.62 | 99.04 |
| L5_2 | 43.69 | 43.2 | 6.48 | 99.25 | 96.02 | 98.88 |
| L5_3 | 43.69 | 43.18 | 6.48 | 99.23 | 95.94 | 98.84 |
| L7_1 | 43.69 | 43.23 | 6.48 | 99.2 | 95.78 | 98.94 |
| L7_2 | 43.69 | 43.17 | 6.48 | 99.28 | 96.19 | 98.81 |
| L7_3 | 43.69 | 43.14 | 6.47 | 99.19 | 95.79 | 98.75 |
| L7R3_1 | 43.69 | 43.23 | 6.48 | 99.23 | 95.92 | 98.94 |
| L7R3_2 | 43.69 | 43.29 | 6.49 | 99.19 | 95.75 | 99.08 |
| L7R3_3 | 43.69 | 43.22 | 6.48 | 99.21 | 95.83 | 98.92 |
| L14_1 | 43.69 | 43.12 | 6.47 | 99.23 | 95.98 | 98.7 |
| L14_2 | 43.69 | 43.22 | 6.48 | 99.24 | 95.98 | 98.91 |
| L14_3 | 43.69 | 43.22 | 6.48 | 99.24 | 95.94 | 98.93 |
| L14R3_1 | 43.69 | 43.11 | 6.47 | 99.3 | 96.34 | 98.68 |
| L14R3_2 | 43.69 | 43.06 | 6.46 | 99.28 | 96.13 | 98.56 |
| L14R3_3 | 43.69 | 43.26 | 6.49 | 99.25 | 95.98 | 99.02 |
| L21_1 | 43.69 | 43.09 | 6.46 | 99.28 | 96.15 | 98.62 |
| L21_2 | 43.69 | 43.16 | 6.47 | 99.18 | 95.73 | 98.79 |
| L21_3 | 43.69 | 43.19 | 6.48 | 99.23 | 95.94 | 98.86 |
| L21R3_1 | 43.69 | 43.21 | 6.48 | 99.19 | 95.73 | 98.89 |
| L21R3_2 | 43.69 | 43.11 | 6.47 | 99.22 | 95.91 | 98.67 |
| L21R3_3 | 43.69 | 42.95 | 6.44 | 99.33 | 96.42 | 98.3 |

Abbreviations used are as follows: D, storage day; R, room temperature storage; L, low temperature storage; Number after R/L: storage days; 1/2/3: three replicates.

**Table S3.** Summary of Genome Mapping for RNA-Seq samples.

| Sample | Total Clean Reads | Total Mapping Ratio | Uniquely Mapping Ratio |
| --- | --- | --- | --- |
| D0_1 | 43.24 | 98.1 | 95.5 |
| D0_2 | 43.14 | 98.07 | 95.48 |
| D0_3 | 43.25 | 98.05 | 95.44 |
| R1_1 | 43.27 | 97.49 | 94.45 |
| R1_2 | 43.14 | 97.47 | 94.49 |
| R1_3 | 43.19 | 97.55 | 94.52 |
| R3_1 | 43.19 | 94.06 | 91.26 |
| R3_2 | 43.19 | 94.17 | 91.32 |
| R3_3 | 43.2 | 94.13 | 91.28 |
| R5_1 | 43.19 | 94.49 | 91.77 |
| R5_2 | 43.27 | 94.68 | 91.9 |
| R5_3 | 43.24 | 94.6 | 91.89 |
| R7_1 | 43.23 | 89.57 | 87.09 |
| R7_2 | 43.15 | 89.58 | 87.01 |
| R7_3 | 43.08 | 89.64 | 87.11 |
| L1_1 | 43.26 | 98.35 | 95.41 |
| L1_2 | 43.23 | 98.45 | 95.48 |
| L1_3 | 43.24 | 98.34 | 95.39 |
| L3_1 | 43.23 | 97.92 | 94.81 |
| L3_2 | 43.27 | 97.95 | 94.88 |
| L3_3 | 43.27 | 97.87 | 94.72 |
| L5_1 | 43.27 | 97.03 | 93 |
| L5_2 | 43.2 | 96.93 | 93 |
| L5_3 | 43.18 | 96.93 | 92.94 |
| L7_1 | 43.23 | 96.7 | 92.21 |
| L7_2 | 43.17 | 96.58 | 92.06 |
| L7_3 | 43.14 | 96.78 | 92.4 |
| L7R3_1 | 43.23 | 91.51 | 88.96 |
| L7R3_2 | 43.29 | 91.51 | 88.85 |
| L7R3_3 | 43.22 | 91.4 | 88.74 |
| L14_1 | 43.12 | 97.84 | 91.78 |
| L14_2 | 43.22 | 97.97 | 91.92 |
| L14_3 | 43.22 | 97.9 | 91.86 |
| L14R3_1 | 43.11 | 93.48 | 91.13 |
| L14R3_2 | 43.06 | 93.57 | 91.2 |
| L14R3_3 | 43.26 | 93.45 | 91.03 |
| L21_1 | 43.09 | 95.44 | 90.44 |
| L21_2 | 43.16 | 95.27 | 90.31 |
| L21_3 | 43.19 | 95.33 | 90.31 |
| L21R3_1 | 43.21 | 94.46 | 91.98 |
| L21R3_2 | 43.11 | 94.52 | 92.02 |
| L21R3_3 | 42.95 | 94.43 | 91.9 |

Abbreviations used are as follows: D, storage day; R, room temperature storage; L, low temperature storage; Number after R/L: storage days; 1/2/3: three replicates.

Table S4. Gene ID in GDR ( https://www.rosaceae.org/) and corresponding gene name in article.

| Gene ID in GDR | Corresponding name in article |
| --- | --- |
| Prupe.1G302700 | *PpPEPC1* |
| Prupe.3G118300 | *PpPEPC2* |
| Prupe.1G039300 | *PpNAD-MDH1* |
| Prupe.4G127500 | *PpNAD-MDH2* |
| Prupe.4G170500 | *PpNAD-MDH3* |
| Prupe.6G212400 | *PpNAD-MDH4* |
| Prupe.2G114100 | *PpNAD-ME1* |
| Prupe.2G160000 | *PpNAD-ME2* |
| Prupe.3G059400 | *PpNADP-ME1* |
| Prupe.3G022700 | *PpAtpvA1* |
| Prupe.6G237200 | *PpAtpvA2* |
| Prupe.3G169600 | *PpAtpvA3* |
| Prupe.1G371400 | *PpAtpvA4* |
| Prupe.8G083200 | *PpAtpvA5* |
| Prupe.3G091900 | *PpVp1* |
| Prupe.6G313800 | *PpVp2* |
| Prupe.1G308100 | *PpALMT4* |
| Prupe.4G009500 | *PptDT1* |


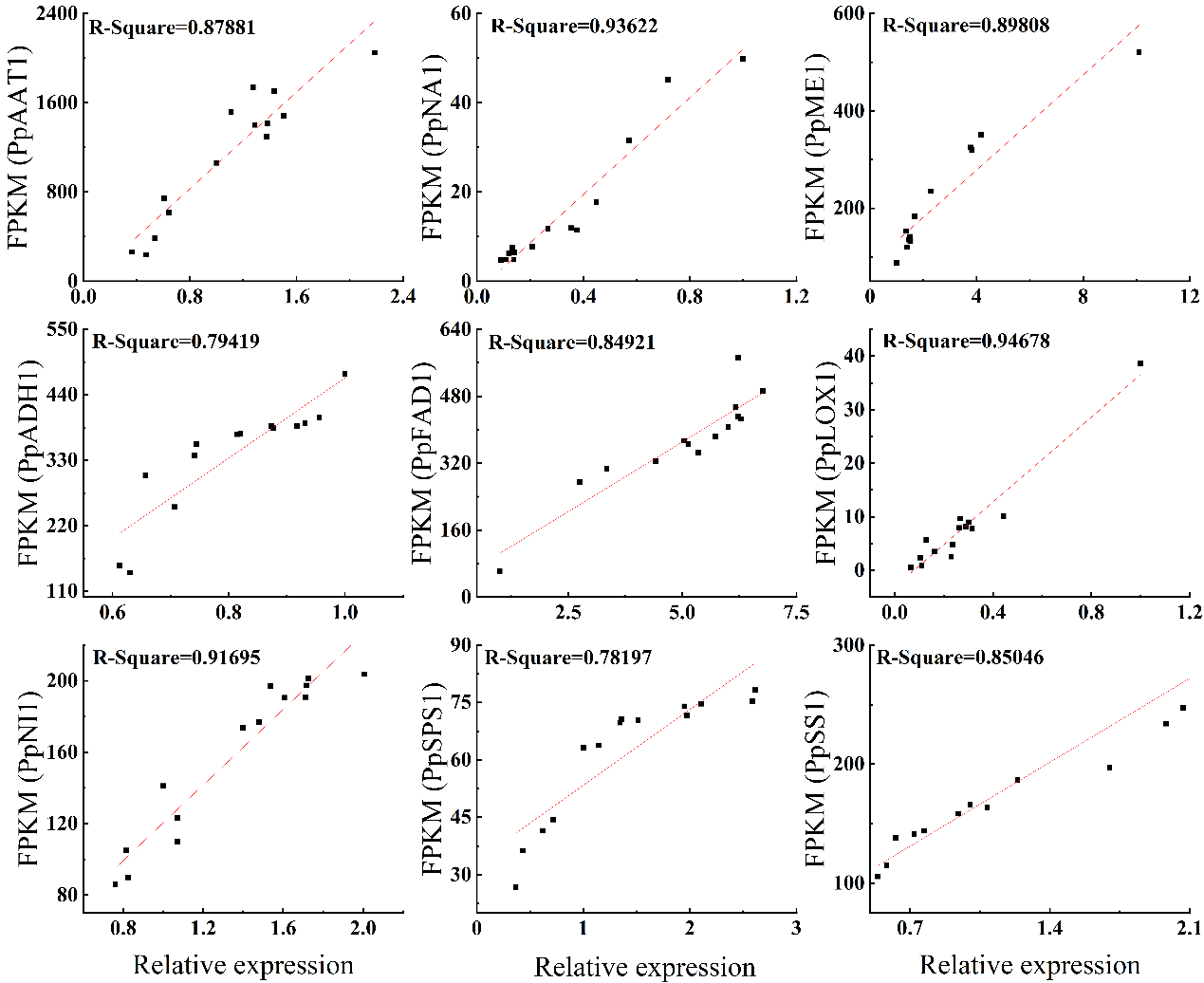


**Figure S1.** The nine expression genes randomly selected were validated through real-time quantitative PCR to assess RNA-Seq data. This comparison relies on relative expression data obtained from real-time quantitative PCR and FPKM values derived from the RNA-Seq results. The line represents the orthogonal fit to the data, with the correlation coefficient (R-Square) displayed. FPKM refers to fragments per kilobase per million mapped fragments.
